# Serotonergic mechanism of the relieving effect of bee venom acupuncture on oxaliplatin-induced neuropathic cold allodynia in rats

**DOI:** 10.1186/1472-6882-14-471

**Published:** 2014-12-06

**Authors:** Ji-Hye Lee, Dong Xing Li, Heera Yoon, Donghyun Go, Fu Shi Quan, Byung-Il Min, Sun Kwang Kim

**Affiliations:** Department of East–west Medicine, Graduate School, Kyung Hee University, Seoul, 130-701 Republic of Korea; Department of Physiology, College of Korean Medicine, Kyung Hee University, Seoul, 130-701 Republic of Korea; Department of Medical Zoology, School of Medicine, Kyung Hee University, Seoul, 130-701 Republic of Korea; Department of Physiology, School of Medicine, Kyung Hee University, Seoul, 130-701 Republic of Korea

**Keywords:** Bee venom acupuncture, Cold allodynia, Oxaliplatin, Rat, Serotonin

## Abstract

**Background:**

Oxaliplatin, an important chemotherapy drug for advanced colorectal cancer, often induces peripheral neuropathy, especially cold allodynia. Our previous study showed that bee venom acupuncture (BVA), which has been traditionally used in Korea to treat various pain symptoms, potently relieves oxaliplatin-induced cold allodynia in rats. However, the mechanism for this anti-allodynic effect of BVA remains poorly understood. We investigated whether and how the central serotonergic system, a well-known pathway for acupuncture analgesia, mediates the relieving effect of BVA on cold allodynia in oxaliplatin-injected rats.

**Methods:**

The behavioral signs of cold allodynia in Sprague–Dawley (SD) rats were induced by a single injection of oxaliplatin (6 mg/kg, i.p.). Before and after BVA treatment, the cold allodynia signs were evaluated by immersing the rat’s tail into cold water (4°C) and measuring the withdrawal latency. For BVA treatment, a diluted BV (0.25 mg/kg) was subcutaneously administered into Yaoyangguan (GV3) acupoint, which is located between the spinous processes of the fourth and the fifth lumbar vertebra. Serotonin was depleted by a daily injection of DL-p-chlorophenylalanine (PCPA, 150 mg/kg, i.p.) for 3 days. The amount of serotonin in the spinal cord was measured by ELISA. Serotonergic receptor antagonists were administered intraperitoneally or intrathecally before BVA treatment.

**Results:**

The serotonin levels in the spinal cord were significantly increased by BVA treatment and such increase was significantly reduced by PCPA. This PCPA pretreatment abolished the relieving effect of BVA on oxaliplatin-induced cold allodynia. Either of methysergide (mixed 5-HT_1_/5-HT_2_ receptor antagonist, 1 mg/kg, i.p.) or MDL-72222 (5-HT_3_ receptor antagonist, 1 mg/kg, i.p) blocked the anti-allodynic effect of BVA. Further, an intrathecal injection of MDL-72222 (12 μg) completely blocked the BVA-induced anti-allodynic action, whereas NAN-190 (5-HT_1A_ receptor antagonist, 15 μg, i.t.) or ketanserin (5-HT_2A_ receptor antagonist, 30 μg, i.t.) did not.

**Conclusions:**

These results suggest that BVA treatment alleviates oxaliplatin-induced acute cold allodynia in rats via activation of the serotonergic system, especially spinal 5-HT_3_ receptors. Thus, our findings may provide a clinically useful evidence for the application of BVA as an alternative therapeutic option for the management of peripheral neuropathy, a dose-limiting side effect that occurs after an administration of oxaliplatin.

## Background

Oxaliplatin, a third-generation platinum-based chemotherapy agent, is considered a central component in the treatment of advanced colorectal cancer [1, 2]. The most important side effect of oxaliplatin treatment is a peripheral neuropathy that has unique characteristics and represents a major dose-limiting toxicity [3, 4]. This unpleasant acute neurosensory toxicity with dysesthesia of the distal extremities and perioral region occurs shortly after an infusion in as much as 90% of the patients. These symptoms can be worsened or triggered by cold [5–7]. However, the mechanisms and the effective treatment for oxaliplatin-induced cold allodynia still remain to be elucidated [8]. Therefore it is worth searching for potential therapeutic options for the management of oxaliplatin-induced neuropathic pain and revealing their action mechanisms.

Bee venom acupuncture (BVA), a kind of chemical stimulation of peripheral nerves (i.e. a subcutaneous injection of diluted BV into one or more acupoints) [9], has long been used in Korea, to relieve pain and to treat inflammatory diseases such as rheumatoid arthritis and osteoarthritis [10–12]. Several animal studies have demonstrated that the analgesic effects of BVA are mediated mainly by activation of α2-adrenergic and/or serotonergic receptors in various pain models, such as nerve injury-induced neuropathic pain, acetic acid-induced visceral pain and inflammatory pain [12–15]. In our recent study [16], we showed that BVA (0.25 mg/kg) treatment at Yaoyangguan (GV3) acupoint significantly attenuated oxaliplatin-induced cold allodynia in rats, which was greater than BVA treatment at the other well-known acupoints for acupuncture analgesia (e.g. Zusanli [ST36] and Quchi [LI11]). However, this anti-allodynic action of BVA was only partially mediated by the noradrenergic system [16]. In addition, the endogenous opioid system that plays a key role in mediating electroacupuncture (EA)-induced anti-allodynia in oxaliplatin-injected rats [17] was not involved in such BVA effect [16]. These results suggest that another analgesic system might be responsible for the relieving effect of BVA on oxaliplatin-induced neuropathic cold allodynia.

The present study was performed to examine whether the suppressive effect of BVA on cold allodynia in oxaliplatin-injected rats is mediated by the serotonergic inhibitory system, which has been regarded as one of the major non-opioid anti-allodynic pathway for acupuncture or EA [18, 19]. We further investigated which subtype of serotonergic receptors is specifically implicated in BVA-induced anti-allodynic action. We report here that BVA treatment exerts its strong relieving effect on oxaliplatin-induced cold allodynia mainly via the activation of spinal 5-HT_3_ receptors, providing a basic evidence for the use of BVA treatment in the management of peripheral neuropathy in oxaliplatin-administered subjects.

## Methods

### Animals

Young adult male Sprague–Dawley rats [Sam:TacN(SD)BR, 200-220 g, 7 weeks old] were housed in cages (3–4 rats per cage) with water and food available ad libitum. The room was maintained with a 12 h-light/dark cycle (a light cycle; 08:00–20:00, a dark cycle; 20:00–08:00) and kept at 23 ± 2°C. All animals were acclimated in their cages for 1 week prior to any experiments. All procedures involving animals were approved by the Institutional Animal Care and Use Committee of Kyung Hee University (KHUASP(SE)-14-010) and were conducted in accordance with the guidelines of the International Association for the Study of Pain [20].

### Oxaliplatin injection

As described previously [21, 22], oxaliplatin (Sigma, St Louis, MO, USA) was dissolved in a 5% glucose (Sigma) solution at a concentration of 2 mg/mL and was intraperitoneally administered at 6 mg/kg. The same volume of 5% glucose solution was injected in the vehicle control group.

### Behavioral test

To estimate whether cold allodynia was induced, cold immersion test was carried out as described previously [18, 23, 24]. Briefly, each animal was lightly immobilized in a plastic holder and its tail was drooped for a proper application of cold water stimuli. The rats were adapted to the holder for 2 days before starting behavioral tests. The tail was immersed in 4°C water, and then the tail withdrawal latency (TWL) was measured with a cut-off time of 15 seconds. The cold immersion test was repeated five times at 5 min intervals. When calculating the average latency, the cut-off time was assigned to the normal responses. The average latency was taken as a measure for the severity of cold allodynia; a shorter TWL was interpreted as more severe allodynia.

Because our previous study [17] showed that a significant cold allodynia sign is induced from 3 days after a single oxaliplatin (6 mg/kg, i.p.) injection and lasted up to 1 week after an injection, we tested whether and how the serotonergic system mediates the relieving effect of BVA on cold allodynia from 3 to 7 days after an oxaliplatin administration. In general, we replicated the experiments twice.

### BVA treatment

We previously showed that the optimal acupoint of BVA-induced anti-allodynia in oxaliplatin-injected rats is Yaoyangguan (GV3) acupoint, and that low doses of BVA (0.25 mg/kg and 1.0 mg/kg) at GV3 is more effective than a high dose of BVA (2.5 mg/kg) [16]. Thus, in this study, BV (0.25 mg/kg) dissolved in normal saline (N/S, 0.05 cc) was injected subcutaneously at GV3 acupoint, which is located between the spinous processes of the fourth and the fifth lumbar vertebrae [25]. To the control group, only 0.05 cc of N/S was injected subcutaneously at the same acupoint.

After baseline cold sensitivity was measured, BV (0.25 mg/kg) and N/S were injected subcutaneously at GV3, respectively. The cold immersion test was performed again at 30 min after BVA.

### Depletion of serotonin

Rats were intraperitoneally administered with DL-p-chlorophenylalanine (PCPA, Sigma, an inhibitor of serotonin synthesis, 150 mg/kg/day) or vehicle (N/S) for three days. The dosage and treatment course of PCPA has been widely used to deplete 5-HT stores [26, 27]. On the day after the final injection of PCPA, oxaliplatin was administerd as described.

### Antagonists

Oxaliplatin-injected rats were divided randomly into four groups: N/S + BV (*n* = 7), DMSO + BV (*n* = 4), methysergide + BV (*n* = 7), and MDL-72222 + BV (*n* = 7). Methysergide maleate (mixed 5-HT_1_/5-HT_2_ receptor antagonist) and 3-tropanyl-3,5-dichlorobenzoate (MDL-72222, 5-HT_3_ receptor antagonist) were dissolved in N/S or 20% dimethyl sulfoxide (DMSO) to a concentration of 1 mg/ml. After baseline cold sensitivity was checked, the four groups were treated intraperitoneally with N/S, 20% DMSO, methysergide (1 mg/kg) and MDL-72222 (1 mg/kg), respectively. Twenty minutes later, all groups were treated subcutaneously with 0.25 mg/kg of BV at GV3 acupoint. The cold immersion test was performed again 30 min after BVA.

To further identify which spinal serotonergic receptor subtypes mediate BVA-induced anti-allodynia, oxaliplatin-injected rats in a different set of experiments were divided randomly into five groups: N/S + BV (*n* = 7), DMSO + BV (*n* = 3), NAN-190 + BV (*n* = 8), ketanserin + BV (*n* = 8), and MDL-72222 + BV (*n* = 8). Ketanserin tartrate (5-HT_2A_ receptor antagonist, 30 *μ*g) was dissolved in 50 *μ*l N/S. 1-(2-methoxyphenyl)-4-(4-[2-phthalimido]butyl) piperazine hydrobromide (NAN-190, 5-HT_1A_ receptor antagonist, 15 *μ*g), and MDL-72222 (12 *μ*g) were dissolved in 50 *μ*l 20% DMSO. Drugs were administered intrathecally under isoflurane anesthesia with the direct lumbar puncture as described previously [28]. Briefly, 50 *μ*l of an antagonist was injected through the L3-L4 intervertebral space, which successfully delivers the drugs to the lower lumbar and sacral spinal cord segments. BVA was performed at 10 min after the intrathecal injection of an antagonist and the cold immersion test was performed again 30 min after BVA.

All drugs were obtained from Tocris Cookson, UK. The doses of antagonists were selected based on previously published studies that show selectivity for the individual receptor subtypes [18, 29–32].

### ELISA

To investigate whether BV treatment increases the quantity of serotonin and whether PCPA decreases that, rats were divided randomly into three groups: Vehicle + N/S, Vehicle + BV, and PCPA + BV (*n* = 5/group). All the animals having oxliplatin-induced cold allodynia signs were sacrificed 30 minutes after BV or N/S treatment, and the lumbar spinal cords of L4 ~ L5 were extracted out on an ice-cold Petri dish as per Glowinski and Iversen [33] and stored at −70°C until the day of analysis. The amount of total protein in the spinal cord samples was measured with the PCA protein assay (PIERCE, USA). Serotonin contents were quantified with the rat 5-HT ELISA kit (LDN, Germany: Catalogue No. BA E-5900; Sensitivity, 0.005 ng/ml; Specificity, 100% cross-reactivity for serotonin) according to the manufacturer’s instructions.

### Statistical analysis

All the data are presented as mean ± SEM. Statistical analysis was done with Prism 5.0 (Graph Pad Software, USA). Paired *t*-test or one-way ANOVA followed by Dunnett’s multiple comparison test was used for statistical analysis. In all cases, *p* < 0.05 was considered significant.

## Results

### Effects of 5-HT depletion with PCPA on BVA-induced anti-allodynia in oxaliplatin-injected rats

**Figure 1:**
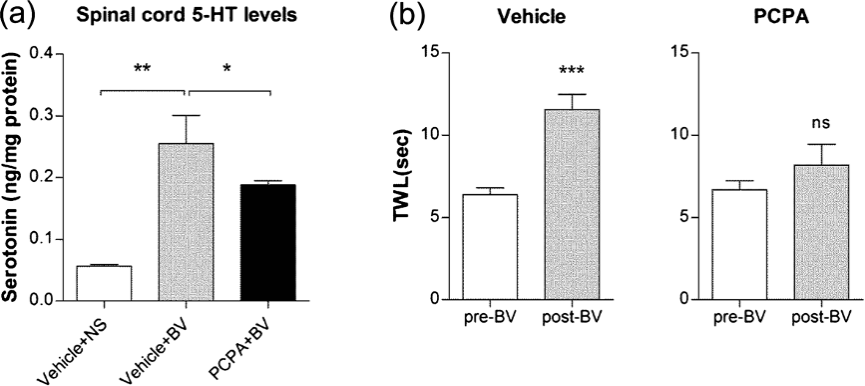
**Effects of PCPA on BVA-induced increases in 5-HT and TWL in oxaliplatin-injected rats. (a)** Levels of 5-HT in the spinal cord measured with ELISA. Vehicle + N/S, Vehicle + BV, and PCPA + BV (*n* = 5/group). Data are presented as mean ± SEM. *p < 0.05, **p < 0.01, by one-way ANOVA followed by Dunnet’s test. **(b)** PCPA, but not vehicle pretreatment (*n* = 8/group), blocked the significant increase in TWL by BVA at GV3 acupoint. Data are presented as mean ± SEM. ***p < 0.001, ns = no significant, by paired t-test.

To determine whether the serotonergic system is involved in the anti-allodynic effect of BVA, we evaluated the effect of depletion of central 5-HT by PCPA on the BVA effect. As shown in Figure 1a, the levels of 5-HT in the spinal cord measured by ELISA were significantly increased after BVA treatment at GV3 (*p* < 0.01, Vehicle + N/S vs. Vehicle + BV). This marked increase in spinal 5-HT levels by BVA was significantly blocked by PCPA pretreatment (*p* < 0.05, Vehicle + BV vs. PCPA + BV). We also confirmed the depletion of 5-HT in the hypothalamus by PCPA (data not shown). These results indicate that BVA treatment markedly increases the amount of central 5-HT, especially in the spinal cord, and PCPA prevents such 5-HT increase by BVA.

Figure 1b shows the effect of 5-HT depletion on the relieving effect of BVA on cold allodynia in oxaliplatin-injected rats. The control group (vehicle (N/S) pretreatment) showed a significant increase in TWL after BVA treatment (*p* < 0.001), whereas the depletion of 5-HT by PCPA pretreatment blocked the anti-allodynic effect of BVA (*p* > 0.05). These results suggest that the serotonergic system plays a major role in mediating the analgesic effect of BVA on oxaliplatin-induced cold allodynia.

### Effects of serotonergic receptor antagonists on BVA-induced anti-allodynia in oxaliplatin-injected rats

**Figure 2:**
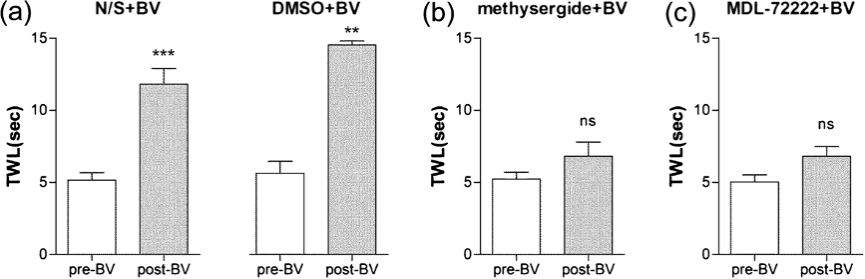
**Effects of systemic injection of 5-HT antagonists on BVA-induced anti-allodynic action.** The behavioral tests for cold allodynia were performed before an intraperitoneal injection of antagonists and after BVA treatment in the four groups of animals: **(a)** N/S + BV (left, *n* = 7) and DMSO + BV (right, *n* = 4), **(b)** methysergide + BV, and **(c)** MDL-72222+ BV (*n* = 7/group). Data are presented as mean ± SEM. **p < 0.01, ***p < 0.001, ns = no significant, by paired t-test.

To investigate which subtype of serotonergic receptors is specifically implicated in the relieving effect of BVA on oxaliplatin-induced cold allodynia, we first tested the effects of a systemic injection of methysergide (mixed 5-HT_1_ /5-HT_2_ receptor antagonist, 1 mg/kg, i.p.) or MDL-72222 (5-HT_3_ receptor antagonist, 1 mg/kg, i.p.) on BVA-induced anti-allodynia. As shown in Figure 2, the N/S or 20% DMSO control group showed a significant increase in TWL after BVA treatment (*p* < 0.01), whereas both experimental groups (methysergide or MDL-72222 pretreated group) exhibited no significant difference in TWL before and after BVA (*p* > 0.05).

**Figure 3:**
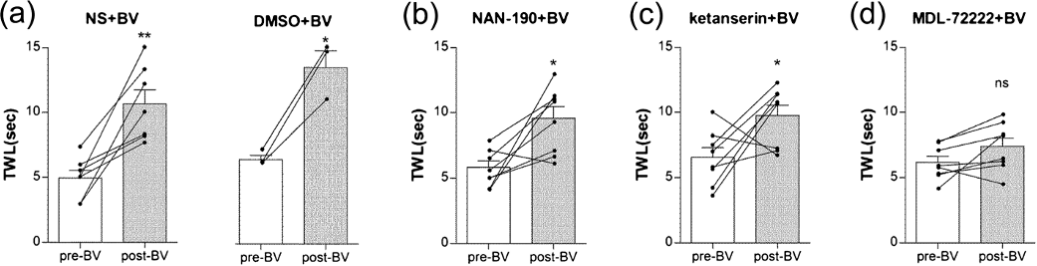
**Effects of intrathecal injection of 5-HT antagonists on BVA-induced anti-allodynic action.** The behavioral tests for cold allodynia were performed before an intrathecal injection of antagonists and after BVA treatment in the five groups of animals: **(a)** N/S + BV (left, *n* = 7) and DMSO + BV (right, *n* = 3), **(b)** NAN-190 + BV, **(c)** ketanserin + BV, and **(d)** MDL-72222 + BV (*n* = 8/group). Data are presented as mean ± SEM (bar graph) and individual traces in TWL change. *p < 0.05, **p < 0.01, ns = no significant, by paired t-test.

We further confirmed that an intrathecal injection of MDL-72222 (12 *μ*g) completely blocked the BVA-induced anti-allodynic action in oxaliplatin-injected rats (*p* > 0.05, Figure 3d). However, as similar to the N/S or 20% DMSO control group, an intrathecal administration of NAN-190 (5-HT_1A_ receptor antagonist, 15 *μ*g) or ketanserin (5-HT_2A_ receptor antagonist, 30 *μ*g) did not block the anti-allodynic effect of BVA (Figure 3a-c). These results suggest that BVA alleviates cold allodynia in oxaliplatin-injected rats mainly via the activation of spinal 5-HT_3_ receptors.

## Discussion

Oxaliplatin, an important chemotherapy drug active against colorectal cancer [1, 2], induces peripheral neuropathy triggered or aggravated in cold conditions even after a single administration [21, 22]. There are few researches showing the effective treatment for the established neuropathic pain induced by oxaliplatin [8]. The potential therapeutic options are thus critically needed to manage this neuropathic pain for helping the patients overcome cancer and improving their quality of life. Our previous study demonstrated for the first time that BVA has a potent relieving effect on oxaliplatin-induced cold allodynia in rats.

Unlike the EA-induced analgesia mainly involving the endogenous opioid system, the analgesic effects of BVA are known to be mediated by the non-opioid, descending noradrenergic system [12, 15, 34, 35]. We recently demonstrated that EA significantly attenuates oxaliplatin-induced cold allodynia in rats via the activation of the endogenous opioids, not the noradrenergic system [17]. However, the anti-allodynic effect of BVA in the same rat model was shown to be only partially mediated by the noradrenergic analgesic system [16]. Therefore, the neurochemical mechanism of the BVA effect on oxaliplatin-induced neuropathic pain still remains unclear.

In the present study, we focused on the other non-opioid analgesic pathway, the serotonergic inhibitory system. This serotonergic system is one of the well-known non-opioid mediators for acupuncture or EA analgesia [19] and a study by Lee and his colleagues reported that the descending serotonergic system is also involved in the antinociceptive effect of BVA in the rat formalin pain model [14]. In the present study, we observed that BVA treatment significantly increases the levels of spinal 5-HT and the TWL in oxaliplatin-injected rats. Such BVA effects were blocked by PCPA depletion of 5-HT (Figure 1). This implies that BVA exerts a potent analgesic effect on oxaliplatin-induced cold allodynia by activating the spinal serotonergic inhibitory system.

It has been known that spinal administration of 5-HT produces analgesic effects, depending on the receptor type activated and dosage use [36–38]. And the analgesic effect of serotonin is reported to be mediated by the descending pain inhibitory system from periaqueductal grey, nucleus raphe magnus and finally spinal serotonergic receptors [39, 40]. Among the several subtypes of serotonin receptors (5-HT_1–7_), 5-HT_1_, 5-HT_2_ and 5-HT_3_ receptors are known to be the most commonly implicated in spinal pain processing [32, 41, 42]. In this study, a systemic injection of methysergide (mixed 5-HT_1_/5-HT_2_ receptor antagonist) or MDL-72222 (5-HT_3_ receptor antagonist) prevented the relieving effect of BVA on cold allodynia (Figure 2). Our additional experiments (Figure 3) further showed that the BVA effect was blocked only by an intrathecal injection of MDL-72222, but not by an intrathecal injection of NAN-190 (5-HT_1A_ receptor antagonist) or ketanserin (5-HT_2A_ receptor antagonist). The discrepancy in the results between systemic and intrathecal administration of antagonists might be due to the use of non-selective antagonist, methysergide for the systemic injection experiment. Also, individual differences in the sensitivity of the rats to intrathecal NAN-190 or ketanserin might contribute to this discrepancy. As shown in Figure 3b and c, the individual changes in TWL before and after BVA treatment in NAN-190 or ketanserin pre-treated rats might be divided into two distinct clusters; three responsive rats and five non-responsive rats to either of the two antagonists. However, both of a systemic injection and an intrathecal injection of MDL-72222 similarly blocked the BVA anti-allodynic effect (Figure 3d). This result is in consistent with the other studies showing that spinal 5-HT_3_ receptors have antinociceptive roles [43–45]. Thus, our data strongly suggest that spinal 5-HT_3_ receptors play an important role in the BVA-induced anti-allodynic action in oxaliplatin-injected rats.

## Conclusions

In conclusion, this study clearly demonstrates a key role of the serotonergic inhibitory system in the relieving effect of BVA on oxaliplatin-induced neuropathic cold allodynia, and specifically, spinal 5-HT_3_ receptors are activated to exert the anti-allodynic effect of BVA. These results provide a basic evidence for the application of BVA as an alternative therapeutic option for the management of oxaliplatin-induced peripheral neuropathy and may raise the possibility of the combinational use of BVA with well-known analgesics, such as morphine, gabapentin and antidepressants.
